# Trends and antibiotic susceptibility patterns of diarrhoeal pathogens – experience over 14 years in southern India

**DOI:** 10.1099/acmi.0.000818.v3

**Published:** 2024-09-23

**Authors:** Ankita Mohanty, Nayannika Lakra, Jharna Mandal

**Affiliations:** 1 Department of Microbiology, JIPMER, Puducherry, 605006, India

**Keywords:** antimicrobials, changing pattern, diarrhoea

## Abstract

**Introduction**. Enteric pathogens contribute significantly to morbidity in a developing country such as India. Early and prompt diagnosis of diarrhoeal diseases can reduce the mortality rate, particularly in children. The pattern of sensitivity to antimicrobials for the common pathogens can vary from time to time. The present study was conducted to study the pathogen distribution and antimicrobial susceptibility pattern during the study period (January 2010 to December 2023).

**Hypothesis/gap statement**. Studying the changing trend in the antimicrobial sensitivity pattern of diarrhoeal pathogens over a decade can help to plan future treatment options.

**Aim**. This study was undertaken to provide insights into the changing pattern of pathogen distribution and antimicrobial susceptibility for enteric pathogens over 14 years.

**Methods**. A retrospective observational cohort analysis was conducted on all the stool pathogens isolated from the samples received in the microbiology department of a tertiary care hospital from 2010 to 2023. The demographic details, stool microscopy, culture reports, and antimicrobial susceptibility patterns were noted.

**Results**. A total of 18 336 stool specimens were received in the microbiology laboratory between January 2010 and December 2023, of which 1354 specimens had diarrhoeal pathogens grown in culture. Out of these 1354 specimens, 591 (44%) had *Salmonella*, 471 (35%) *Shigella*, 181 (13%) *Vibrio cholerae,* and 80 (6%) *Aeromonas* species. Among these pathogens, susceptibility to ceftriaxone was seen in 93% (552 isolates) of *Salmonella* species, 89% (420 isolates) of *Shigella* species, and 95% (171 isolates) of *Vibrio cholerae*; 91% (73 isolates) of *Aeromonas* species were susceptible to chloramphenicol. Some major parasites were also observed on microscopy.

**Conclusion**. Timely diagnosis of diarrhoeal pathogens can be life-saving for patients at the extremes of age, i.e. in children and the elderly. Pathogens can exhibit a changing susceptibility pattern to antibiotics, which should be regularly observed to plan future therapy.

## Data Summary

All data associated with this work are reported within the article.

## Introduction

Gastroenteritis is considered to be an important health problem in India. It is a condition caused by bacteria, viruses, and parasites [1] and is a major cause of illness and death in various age groups, especially at the extremes of age groups, i.e. in children and the elderly [2]. This can be attributed to poor sanitary conditions, improper personal hygiene, and lack of proper homes. Natural calamities such as floods, cyclones, and rainstorms can worsen the situation. Nearly 90% of clinical cases of gastroenteritis are due to polluted drinking water, improper sanitation, and poor hygiene [3], associated with an ever-increasing multidrug resistance among diarrhoeal pathogens due to the indiscriminate use of antimicrobials. Ironically, amidst the rise and outbreak of resistant strains, the emergence of strains susceptible to older antibiotics has been noted [4]. Most diarrhoea cases can be managed with hydration and antibiotics are only indicated in certain situations and should be selected appropriately for the target pathogen [5]. It is important to study the distribution of enteric pathogens and their antibiotic susceptibility pattern over time in a geographical area to collect information about the common pathogens in that area. This can help formulate the antibiotic policy for empirical therapy and prevent complications by providing early treatment. Traditionally, fluoroquinolones have been used for treating diarrhoea, but due to the development of resistance, azithromycin and doxycycline have replaced them. Third-generation cephalosporins are currently considered the drug of choice for *Shigella* and *Salmonella* species, while doxycycline is the drug of choice for for *Vibrio cholerae*. The current study aims to study the organism profiles and antibiograms of the isolates from stool samples from cases of diarrhoea/dysentery in a tertiary care hospital. This study highlights the emerging trend in the pathogen distribution and antibiotic susceptibility of diarrhoeal isolates as well as stool parasites from 2010 to 2023.

## Methods

### Study design

A retrospective observational cohort analysis was conducted on all the stool pathogens isolated from the samples received from January 2010 to December 2023 with approval from the Institutional Ethics Committee (JIP/IEC/2017/0130).

### Study setting

This study was conducted in a tertiary care hospital, located in the southern part of India.

### Data collection

The data were collected retrospectively from the laboratory records and the laboratory information system. The demographic details, clinical history of the patients having significant microscopic findings, and pathogens in the stool samples were recorded. A standard protocol for stool processing for culture was used, employing a selective medium (xylose lysine deoxycholate agar), less selective cum differential medium (MacConkey agar), and an enrichment broth (selenite F broth); suspected colonies were screened using catalase test, oxidase test, indole detection, citrate, urea, Kligler iron agar, lysine iron agar, and mannitol motility medium. In the case of suspected cholera cases, thiosulphate citrate bile salt sucrose agar (TCBS) was also used and the specimen was inoculated into alkaline peptone water (APW). Antibiotic sensitivity testing was performed using the Kirby–Bauer technique for a panel of antibiotics (ampicillin, ceftriaxone, cotrimoxazole, cefixime, chloramphenicol, tetracycline, ciprofloxacin) and interpreted as per the CLSI M100 guidelines for *Salmonella* and *Shigella* and the CLSI M45 guidelines for *Vibrio* and *Aeromonas* for the respective years. The bacterial profile and antimicrobial susceptibility pattern of the bacteria, namely *Salmonella* species, *Vibrio* species, *Shigella* species, and *Aeromonas* species, was noted. *Plesiomonas shigelloides*, *Edwardsiella tarda*, *Comamonas aquatica,* and *Yersinia enterocolitica* were also included. Identification of *Salmonella* species*, Vibrio* species*, Aeromonas* species, *P. shigelloides*, *E. tarda*, and *C. aquatica* was simplified after the introduction of MALDI-TOF MS (bioMérieux, France) in 2019.

Serogrouping of *Shigella, Salmonella* and *V. cholerae* was performed using corresponding antisera (BD Diagnostics). Parasites detected were also noted.

### Statistical analysis

The extended Mantel–Haenszel chi-square test was applied to observe the significance in the various age groups considering the absolute count of the organisms present and absent in a particular age group.

Antibiotic sensitivity patterns for the duration of the study period (14 years) are shown as linear trends in the form of line graphs.

## Results

**Fig. 1.**
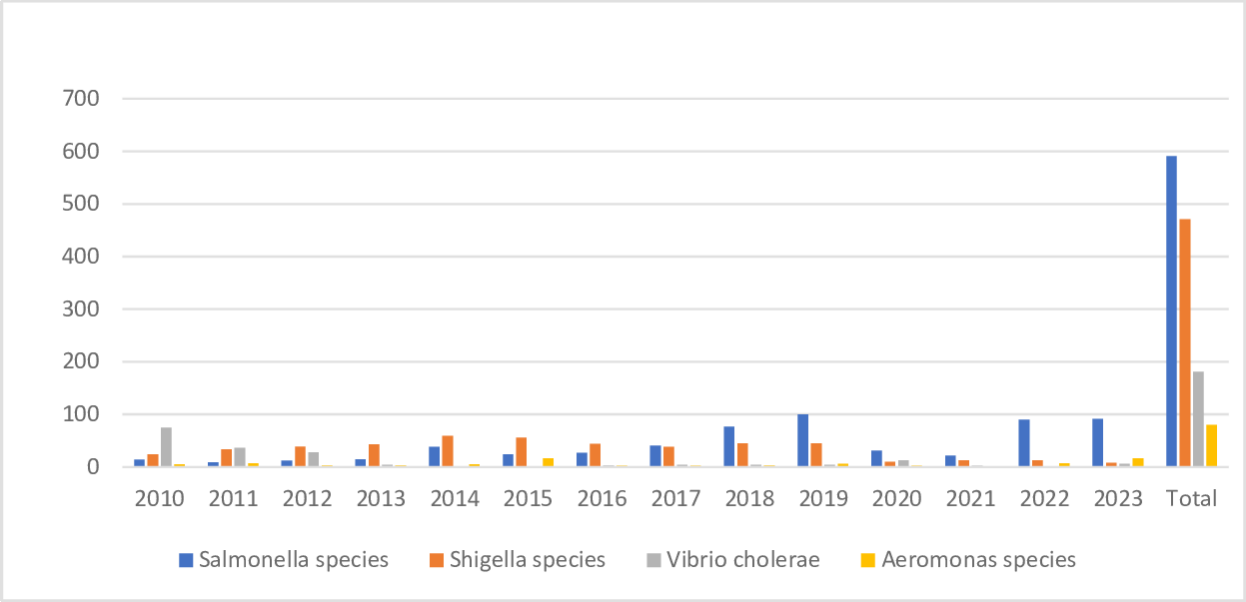
Year-wise distribution of common diarrhoeal pathogens.

A total of 18  336 stool specimens were received in the microbiology laboratory between January 2010 to December 2023. A total of 591 *Salmonella*, 471 *Shigella*, 181 *V. cholerae* and 80 *Aeromonas* organisms were isolated. The pathogen-wise distribution is shown in Table 1 and Fig. 1.

**Table 1.** Year-wise distribution of the common diarrhoeal pathogens

Year	*Salmonella* species	*Shigella* species	*Vibrio cholerae*	*Aeromonas* species
2010	14	24	75	5
2011	9	34	36	7
2012	12	39	28	3
2013	15	43	4	3
2014	38	59	1	5
2015	24	56	0	17
2016	27	44	3	2
2017	41	38	4	2
2018	77	45	4	3
2019	100	45	4	6
2020	31	10	13	2
2021	22	13	2	1
2022	90	13	1	7
2023	91	8	6	17
Total	591	471	181	80

**Fig. 2.**
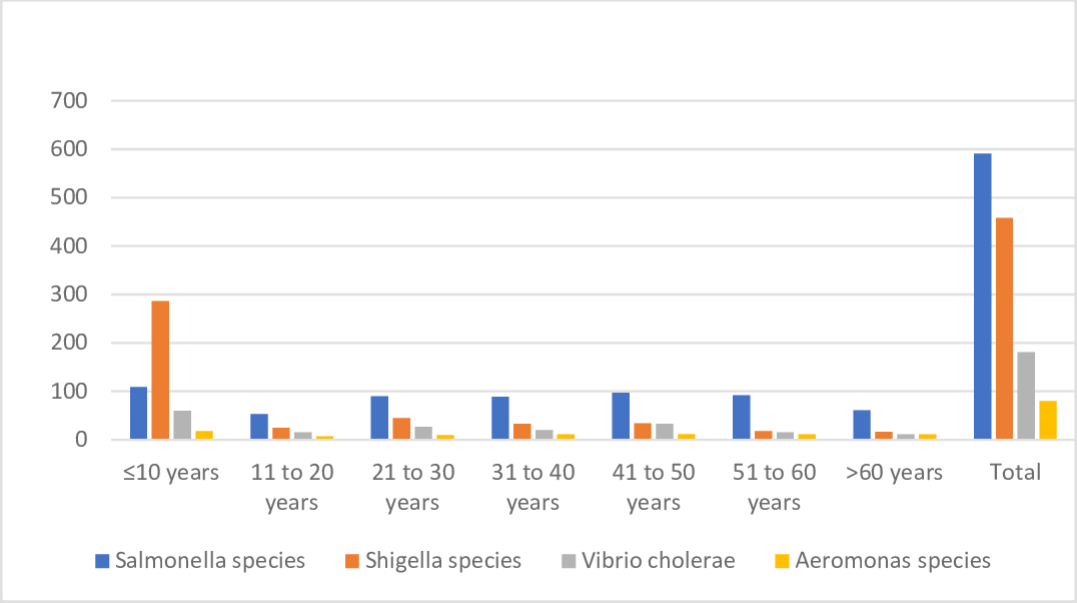
Age-wise distribution of diarrhoeal pathogens.

The age-wise distribution of pathogens is shown in Table 2 and Fig. 2. The trend over the age groups was significant for *V. cholerae* (*P=*0.0000001 by chi-square test) and *Shigella* species (*P=*0.0000001 by chi-square test) while it was insignificant for *Salmonella* and *Aeromonas* species.

**Table 2.** Age-wise distribution of the common diarrhoeal pathogens

Age	*Salmonella* species	*Shigella* species	*Vibrio cholerae*	*Aeromonas* species
≤10 years	109	294	60	18
11–20 years	53	25	15	7
21–30 years	90	47	27	10
31–40 years	89	34	20	11
41–50 years	97	35	33	12
51–60 years	92	19	15	11
>60 years	61	17	11	11
Total	591	471	181	80

**Fig. 3.**
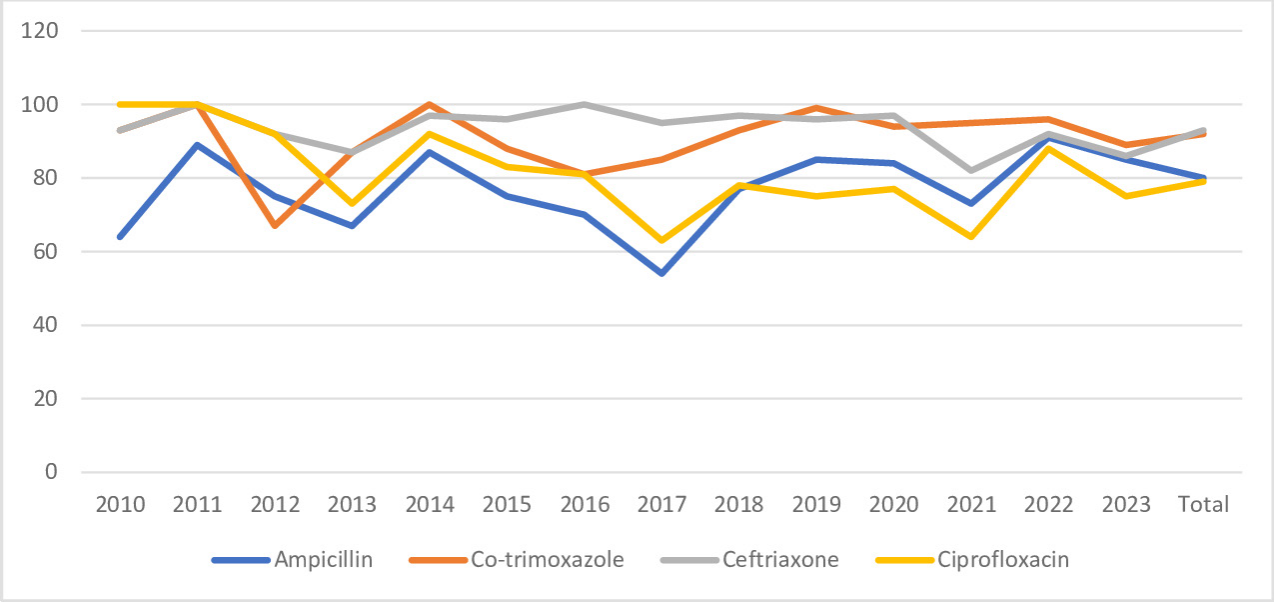
Antibiotic susceptibility pattern of *Salmonella* species.

Most of the *Salmonella* isolates were susceptible to ceftriaxone (93%), with a decreased susceptibility to nalidixic acid and furazolidone (Table 3 and Fig. 3). This was observed in the initial years (2010–2012), following which these antibiotics were abandoned because of increasing resistance.

**Table 3.** Antibiotic susceptibility pattern of *Salmonella* species*

Antibiotics	2010(*n*=14)	2011(*n*=9)	2012(*n*=12)	2013(*n*=15)	2014(*n*=38)	2015(*n*=24)	2016(*n*=27)	2017(*n*=41)	2018(*n*=77)	2019(*n*=100)	2020(*n*=31)	2021(*n*=22)	2022(*n*=90)	2023(*n*=91)	Total (*n*=591)
Ampicillin	64	89	75	67	87	75	70	54	77	85	84	73	91	85	80
Co-trimoxazole	93	100	67	87	100	88	81	85	93	99	94	95	96	89	92
Ceftriaxone	93	100	92	87	97	96	100	95	97	96	97	82	92	86	93
Ciprofloxacin	100	100	92	73	92	83	81	63	78	75	77	64	88	75	79

*Percentage susceptibility values are mentioned in the table.

**Fig. 4.**
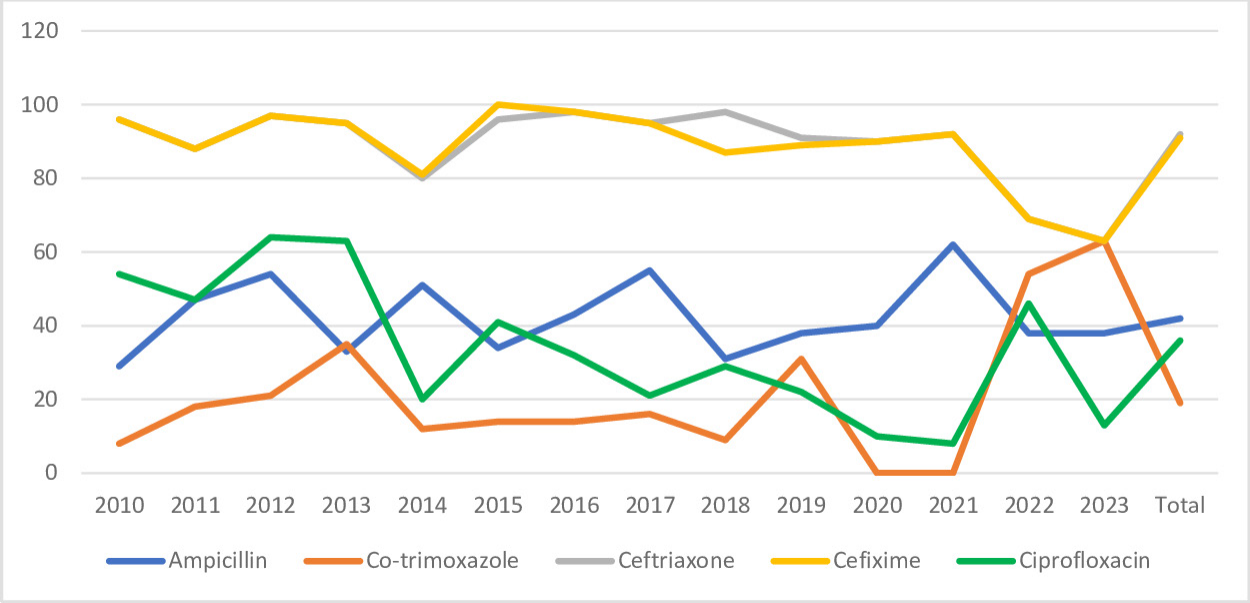
Antibiotic susceptibility pattern of *Shigella* species.

Most of the *Shigella* species were susceptible to ceftriaxone (92%), with a few being resistant to it (Table 4 and Fig. 4).

**Table 4.** Antibiotic susceptibility pattern of *Shigella* species*

Antibiotics	2010(*n*=24)	2011(*n*=34)	2012(*n*=39)	2013(*n*=43)	2014(*n*=59)	2015(*n*=56)	2016(*n*=44)	2017(*n*=38)	2018(*n*=45)	2019(*n*=45)	2020(*n*=10)	2021(*n*=13)	2022(*n*=13)	2023(*n*=8)	Total (*n*=471)
Ampicillin	29	47	54	33	51	34	43	55	31	38	40	62	38	38	42
Co-trimoxazole	8	18	21	35	12	14	14	16	9	31	0	0	54	63	19
Ceftriaxone	96	88	97	95	80	96	98	95	98	91	90	92	69	63	92
Cefixime	96	88	97	95	81	100	98	95	87	89	90	92	69	63	91
Ciprofloxacin	54	47	64	63	20	41	32	21	29	22	10	8	46	13	36

*Percentage susceptibility values are mentioned in the table.

**Fig. 5.**
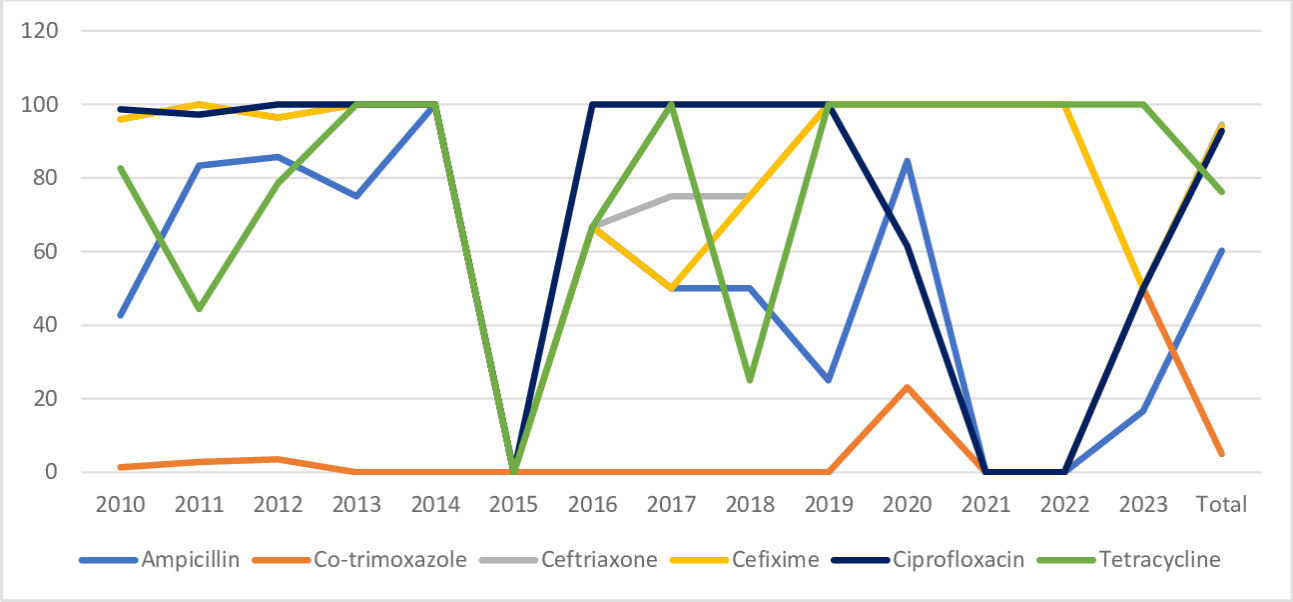
Antibiotic susceptibility pattern of *Vibrio cholerae*.

**Fig. 6.**
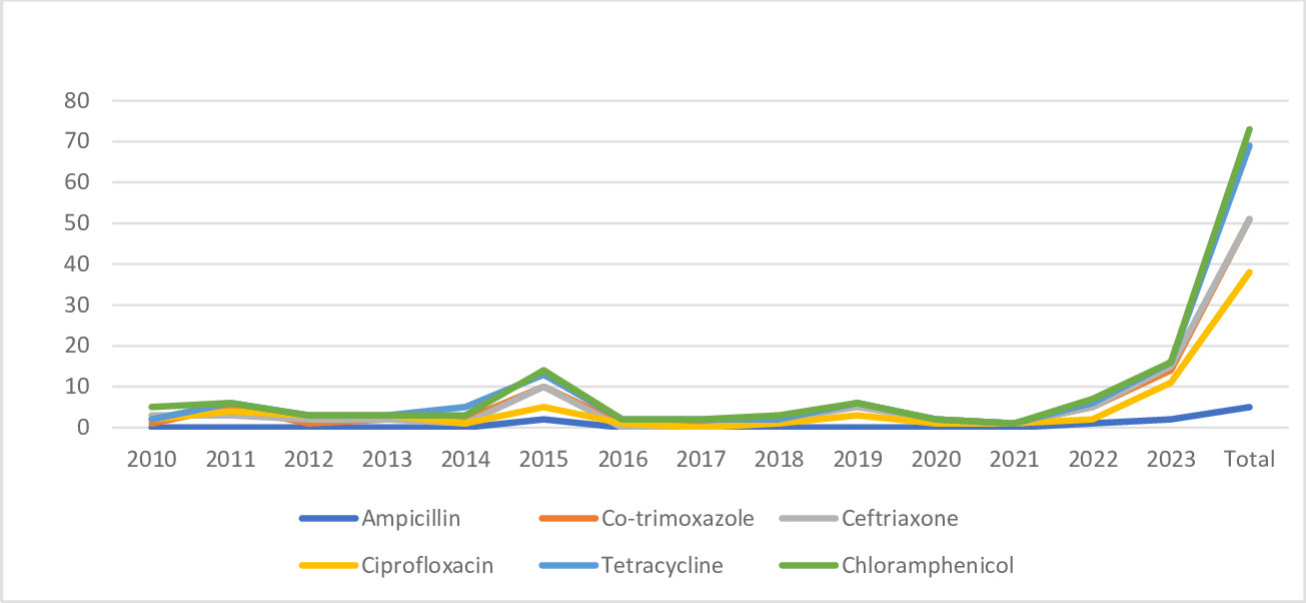
Antibiotic susceptibility pattern of *Aeromonas* species.

Among the *V. cholerae* isolates, the majority were susceptible to ceftriaxone (94%) (Table 5 and Fig. 5), while 91% (73/80) of the *Aeromonas* species were susceptible to chloramphenicol (Table 6 and Fig. 6).

**Table 5.** Antibiotic susceptibility pattern of Vibrio cholerae*

Antibiotics	2010(*n*=75)	2011(*n*=36)	2012(*n*=28)	2013(*n*=4)	2014(*n*=1)	2015(*n*=0)	2016(*n*=3)	2017(*n*=4)	2018(*n*=4)	2019(*n*=4)	2020(*n*=13)	2021(*n*=2)	2022(*n*=1)	2023(*n*=6)	Total (*n*=181)
Ampicillin	43	83	86	75	100	0	67	50	50	25	85	0	0	17	60
Co-trimoxazole	1	3	4	0	0	0	0	0	0	0	23	0	0	50	5
Ceftriaxone	96	100	96	100	100	0	67	75	75	100	100	100	100	50	94
Cefixime	96	100	96	100	100	0	67	50	75	100	100	100	100	50	94
Ciprofloxacin	99	97	100	100	100	0	100	100	100	100	62	0	0	50	93
Tetracycline	83	44	79	100	100	0	67	100	25	100	100	100	100	100	76

*Percentage susceptibility values are mentioned in the table.

**Table 6.** Antibiotic susceptibility pattern of *Aeromonas* species (*n*=80)*

	2010	2011	2012	2013	2014	2015	2016	2017	2018	2019	2020	2021	2022	2023	Total (%)
Ampicillin	0	0	0	0	0	2	0	0	0	0	0	0	1	2	5 (6%)
Co-trimoxazole	1	5	1	2	2	10	1	1	1	6	1	1	5	14	51 (64%)
Ceftriaxone	3	3	2	2	1	10	0	0	2	5	2	1	5	15	51 (64%)
Ciprofloxacin	2	4	3	3	1	5	1	0	1	3	1	1	2	11	38 (48%)
Tetracycline	2	6	3	3	5	13	2	2	2	6	2	1	6	16	69 (86%)
Chloramphenicol	5	6	3	3	3	14	2	2	3	6	2	1	7	16	73 (91%)

*The number of susceptible isolates is mentioned in the table.

In addition to the above-mentioned bacterial pathogens, other bacteria isolated were *Campylobacter* species (4), enteroaggregative *Escherichia coli* (EAEC) (1), enterohemorrhagic *E. coli* (EHEC) (1), *Y. enterocolitica* (1), *E. tarda* (4), *P. shigelloides* (8), *C. aquatica* (10), and *Vibrio vulnificus* (2).

The most commonly observed parasitic elements were cysts or trophozoites of *Entamoeba* spp. (*n*=54) followed by hookworm eggs and larvae (*n*=49), cysts and trophozoites of *Giardia* (*n*=37), *Ascaris lumbricoides* egg (*n*=32) and *Strongyloides* spp. larvae (*n*=29), egg of *Enterobius vermicularis* (*n*=6), *Hymenolepis nana* (*n*=1), and *Trichuris trichiura* (*n*=2).

## Discussion

The present study is a retrospective analysis of the data obtained from 18  336 stool specimens received in the Department of Microbiology from 2010 to 2023. The demographic details, clinical history of the patients having significant microscopic findings, and pathogens in the stool samples were recorded. The bacterial profile and antimicrobial susceptibility pattern of the bacteria were noted. A total of 591 *Salmonella*, 471 *Shigella,* 181 *Vibrio*, and 80 *Aeromonas* species were isolated.

*Salmonella*, *Shigella*, and *Vibrio* were common in children <10 years of age. In a study by Anjeeta *et al.,* the highest occurrence of diarrhoeal disease was reported among children aged 9–12 years (71.4%), while the lowest incidence was seen in the age group 5–8 years (33.3%) [6]. An increase in the incidence of diarrhoeal diseases with an increase in the age of the children was noted. This could be due to improper hand washing habits of the children as well as improper sanitary habits of caretakers after changing the napkins of the children. For most of these isolates, decreased susceptibility to nalidixic acid and furazolidone led to the discontinuation of these drugs for treatment. The trend with the different organisms is discussed subsequently.

### Trends in the *Salmonella* species

Nontyphoidal *Salmonella* (NTS) infections are a common cause of diarrhoeal disease and are responsible for causing 93 million enteric infections and 155 000 diarrhoeal deaths yearly [7]. Most of the NTS are usually associated with self-limiting gastroenteritis, which does not require antimicrobial treatment, but there has been evidence of invasive diseases such as bloodstream infections being caused by them in patients with underlying comorbidities [8]. This necessitates the institution of antimicrobial therapy in cases of immunocompromised individuals with co-morbidities. Chloramphenicol, ampicillin, and trimethoprim/sulphamethoxazole (cotrimoxazole) were the drugs of choice for the treatment of salmonellosis in the past [9]. In our study, resistance to conventional classes of antibiotics such as chloramphenicol, nalidixic acid, and furazolidone was observed. Interestingly, NTS that were initially resistant to cephalosporins and fluoroquinolones have shown a diminishing trend of resistance to these antibiotics in recent years [10], and a similar finding was also observed in our isolates. In a study performed by Jacob *et al.* in southern India, resistance to nalidixic acid (27%) was most common among the tested NTS, followed by resistance to ampicillin (19.3%), cotrimoxazole (14%), ciprofloxacin (12.4%), and chloramphenicol (4%), which is similar to the findings of our study. Ceftriaxone had the highest sensitivity [11]. The resistance to beta-lactam antibiotics such as ceftriaxone in this organism is remarkable. This is caused by extended-spectrum beta-lactamases (ESBLs), predominantly CTX-M-15 and SHV-12, being present in those strains [12,13]. An ACC-1 AmpC *Salmonella* Typhi was identified in the blood culture of a 14-year-old girl from our setting in the past. It could have been acquired from drug-resistant bowel flora and atypical resistance was seen with it [14]. Genotypic diversity has been observed among the strains present in our region by pulsed-field gel electrophoresis, indicating the existence of many different clones of *S.* Typhi universally [15].

### Trends in the *Shigella* species

Shigellosis can be life-threatening in populations at the extremes of age (young, elderly). In most communities, the incidence of shigellosis is higher in the summer months due to reduced hand hygiene habits, but a significant peak is also noted during the rainy season [16]. In our geographical region, *Shigella flexneri* was found to be the most common followed by *Shigella sonnei, Shigella boydii*, and *Shigella dysenteriae* [17]. Cefixime became the drug of choice for shigellosis in our centre from 2012 onwards due to the development of resistance to ciprofloxacin, co-trimoxazole, and ampicillin. Such a resistance pattern points to the ability of *Shigella* to survive and replicate in the human gut and incorporate externally derived genetic material, including antimicrobial resistance (AMR) genes on transposons from other Gram-negative bacteria [18]. With the ease of its availability as well as administration, ciprofloxacin was used by the majority of clinicians, which has contributed to resistance. The pattern of resistance in our study was nalidixic acid followed by cotrimoxazole, ciprofloxacin, ampicillin, and then ceftriaxone, in order of decreasing resistance. We observed a high resistance to ciprofloxacin, as reported in an earlier study from India [19].

The isolates from our centre were also found to harbour extended-spectrum β-lactamases (ESBLs). A study conducted revealed three ceftriaxone-resistant *S. flexneri* strains harbouring CTX-M-15, CTX-M-14, and TEM-1 genes [20]. ESBL-producing *Shigella* were reported from other parts of India, as well as from countries such as the Republic of Korea, Argentina, Vietnam, France, and Turkey [21,26].

Virulence genes have shown diversity in all serotypes of *S. flexneri*. *IpaH* (invasion plasmid antigen H) and *ial* (invasion-associated locus) genes were also detected in almost all of the isolates [17].

### Trends in the *V. cholerae*

Cholera epidemics caused by toxigenic *V. cholerae* pose a major threat in most developing countries [27]. Globally, epidemics of cholera have been reported in 47 countries [28]. Outbreaks were observed during the rainy season.

In Kolkata, India, during two consecutive cholera seasons (1989–1990), the Inaba serotype was prevalent, but in our study period (2010–19) the Ogawa serotype was predominant [29]. Similar genetic conversions for cholera have been reported from other parts of the world.

A comparison of contemporary reports of antibiotic sensitivity patterns for *V. cholerae* strains with those isolated in the past shows a vast difference in the antimicrobial resistance profile. The reversal of the antimicrobial susceptibility pattern is due to the decreased use of conventional drugs that had encountered resistance earlier [30]. In the current study, reduced susceptibility to the earlier drugs, such as co-trimoxazole, was observed. In a study performed in the same setup from 2008 to 2013, the prevalence of SXT element conserved genes (*int, eex, att-prfC,* and *setR*) was noted in all of the isolates. The spread of Haitian-like traits with a creeping MIC (0.75–2 µg m^−1^) for azithromycin was also noted [31]. A study performed in different parts of Odisha from 2004 to 2013 also showed decreased susceptibility to co-trimoxazole [32]. Sensitivity to ciprofloxacin was the highest, making it a drug of choice, and the sensitivity to tetracyclines showed a cyclical trend in our study, similar to a study performed in Bangladesh [33].

A meta-analysis performed by Yuan *et al.* showed a low resistance rate against some antibiotics, including fluoroquinolones, gentamicin, ceftriaxone, doxycycline, kanamycin, and cefotaxime, similar to our study [34]. Antibiotic resistance in *V. cholerae* has been reported to be due to mobile genetic elements and plasmid-mediated resistance. In our study setup, a *V. cholerae* Amp C producer strain due to the *bla*DHA gene and a carbapenemase producer due to the *blaNDM-1* gene had been isolated in the past [35].

### Trends in *Aeromonas* species

*Aeromonas* is an important but often neglected pathogen causing gastrointestinal diseases. It can mimic cholera. There have been several reports of increase in drug-resistant *Aeromonas*, especially with respect to beta-lactams, quinolones, and tetracyclines. In our study, maximum resistance was detected in *Aeromonas* species for ampicillin, though only a few species are known to be intrinsically resistant to it. Many of our isolates were found to be resistant to the third-generation cephalosporins, with the MIC for ceftriaxone being ≥4 µg ml^−1^ [36]. The presence of *bla*CTX-M and inducible AmpC beta-lactamase (presence of MOX gene) have been documented in 70 and 24% of the isolates. Most commonly CTX M-15 is isolated in India. Fluoroquinolones have been the drug of choice but isolates that are resistant to nalidixic acid and susceptible to ciprofloxacin are known to possess the *gyrA* gene and hence can develop resistance to fluoroquinolones [37]. In our study, a cyclical trend was observed for fluoroquinolones. CphA harboured in common *Aeromonas* species such as *A. hydrophila*, *A. dhakensis*, *A. jandaei*, and *A. veronii* is the main chromosomal MBL recognized in aeromonads [38]. Contaminated water sources can act as a medium for the transfer of antimicrobial-resistant genes.

In a study performed by Roman *et al.*, higher susceptibility to tetracyclines was noted for *Aeromonas* species, similar to our study [39]. However, in some Asian studies, tetracycline resistance was also noted [40,42].

In a study performed by Mohan *et al.* in northern India, resistance was observed to fluoroquinolones and co-trimoxazole for *Aeromonas* species, similar to our study [43].

### Heterogeneity of the pathogens

Heterogeneity in terms of serotypes, resistotypes, or genotypes is an important factor observed in our diarrhoeal isolates of *Shigella*, *Salmonella*, and *V. cholerae*, as far as the survival of these pathogens is concerned. The extensive use of azithromycin led to the rise of drug-resistant strains of *S. sonnei* [44], a phenomenon observed in our study as well. Serotype switching among *Shigella* species is a well-known phenomenon and leads to the emergence of many untypable *Shigella* species, as documented in an earlier study [45]. Our currently circulating strains of *S. flexneri* are of serotype 2a, followed by 6 and 3b; though some rare types such as type 1 variant and type 4 were also seen. O antigen loss (OmpA), an observation in a few of our *S. flexneri* strains [17], is another factor that can affect the conventional serotyping and may lead to the failure of an effective vaccine targeted against these antigens. Genetic heterogeneity was detected by CRISPR among our *S. flexneri* isolates; variations were substantial, and CRISPR type 3 was the most predominant [46]. Chen *et al.*, revealed that the presence of multiple insertion sequences in the cas genes lowered the activity of the CRISPR–Cas system, aiding the bacteria to acquire more extra genetic elements such as the AMR genes through horizontal gene transfer [47].

*Salmonella*, on the other hand, is known to show variations in its O and H antigens to escape immune clearance, often leading to either loss of these antigens or their masking, which can affect the conventional serotyping results. The emergence of third-generation-resistant *Salmonella* isolates, both typhoidal and NTS, because of the rampant use of oral cefixime has probably led to this [48].

The major driving force in the heterogenous characteristics of these pathogens is horizontal gene transfer, depending on genomic plasticity, which leads to increasing AMR and selecting out strains/species in response.

### Other bacterial pathogens isolated

*Campylobacter* species-level identification was performed in our setup using multiplex PCR targeting 16S rRNA (genus *Campylobacter*), *mapA* (*C. jejuni*), *ceuA* (*C. coli*), and *actB* for internal control [49]. *E. tarda* infections may be attributed to the presence of a marine environment. *P. shigelloides* has been associated with ingestion of seafood, which is a common practice in coastal areas.

### Bacterial and parasitic co-infections in our study

Some bacterial and parasitic co-infections were observed in our study. *Shigella* and *Salmonella* co-infection with the parasites was commonly noted. Parasites have certain mechanisms that they use to manipulate or evade the host immune response and cause infection [50]. Parasites can alter the host’s immune response and affect susceptibility to other infections. Normal gut flora affected by helminth can trigger the ability of bacteria to invade the intestine [51].

## Conclusion

Diarrhoeal diseases pose a major threat in developing countries. They can be caused by a lack of proper sanitary facilities and access to safe drinking water. Limited access to laboratory facilities in developing countries forces clinicians to seek a syndromic approach and empirical use of broad-spectrum antibiotics, paving the way for the increasing number of drug-resistant strains. Diarrhoeal pathogens such as *Vibrio* and *Aeromonas* are found freely in the environment and can survive in extreme conditions, hence the resistant traits can be passed on to various strains. Contaminated water can act as an agent for the transfer of antimicrobial resistance genes. Early diagnosis, targeted therapy, and proper antibiograms for diarrhoeal diseases can help in the reduction of resistant strains. Proper laboratory facilities for detecting the ESBL-producing strains and knowledge about the resistant genes prevailing in a geographical area can help to combat the spread of antimicrobial resistance among pathogens in the long run.

## Abbreviations

AMR: antimicrobial resistance
CRISPR: clustered regularly interspaced short palindromic repeats
MALDI-TOF MS: matrix-assisted laser desorption/ionization time-of-flight mass spectrometry
NTS: nontyphoidal *Salmonella*
PCR: polymerase chain reaction
